# Intervention with *α*-Ketoglutarate Ameliorates Colitis-Related Colorectal Carcinoma via Modulation of the Gut Microbiome

**DOI:** 10.1155/2019/8020785

**Published:** 2019-06-17

**Authors:** Si Li, Chenxing Fu, Yurong Zhao, Jianhua He

**Affiliations:** College of Animal Science and Technology, Hunan Agricultural University, Changsha, Hunan 410128, China

## Abstract

The intestinal microbiome plays a crucial role in promoting intestinal health, and perturbations to its constitution may result in chronic intestinal inflammation and lead to colorectal cancer (CRC). *α*-Ketoglutarate is an important intermediary in the NF-*κ*B-mediated inflammatory pathway that maintains intestinal homeostasis and prevents initiation of intestinal inflammation, a known precursor to carcinoma development. The objective of this study was to assess the potential protective effects of *α*-ketoglutarate intervention against CRC development, which may arise due to its known anti-inflammatory and antitumour effects. CRC was induced in C57BL/6 mice using azoxymethane (AOM) and dextran sulfate sodium (DSS). Tumour frequency, histological rating, and colonic microbiota were assessed in colonic samples. The findings demonstrated that *α*-ketoglutarate offered significant protection against CRC development in mice. Furthermore, *α*-ketoglutarate also exhibited immunomodulatory effects mediated via downregulation of interleukin (IL)-6, IL-22, tumour necrosis factor (TNF)-*α*, and IL-1*β* cytokines. Finally, intervention with *α*-ketoglutarate tended to minimise the frequency of opportunistic pathogens (*Escherichia* and* Enterococcus*) while increasing the populations of* Akkermansia, Butyricicoccus*,* Clostridium,* and* Ruminococcus*. Taken together, our findings show that dietary *α*-ketoglutarate intervention may protect against inflammation-related CRC.

## 1. Introduction

Colorectal cancer (CRC) represents a significant public health concern, comprising one of the top three most frequent global cancers, with almost 1.2 million new incidences reported annually and with a mortality rate of ~40% [1]. CRC incidence can be correlated with various genetic and environmental influences [2, 3]; for example, most colonic carcinogenesis develops in patients with inflammatory bowel disease (IBD) [4, 5]. CRC has been the subject of many studies and can be subdivided into two varieties: (1) colitis-associated CRC and (2) sporadic CRC. Extended periods of IBD are among the main risk factors associated with colitis-associated CRC initiation. This is typified by colitis-associated cancer incidence presenting at a rate of 2% following a 10-year history of IBD, rising up to 18% following a 30-year IBD history [6].

To understand the relationship between intestinal microbiota and CRC development, a number of animal-based CRC models have been developed in the past few decades. Such models have a basis in both genetic engineering (for example,* Muc2*^−/−^ and IL-10^−/−^ mice) [7, 8] and chemical interventions (for example, azoxymethane [AOM] and/or dextran sulfate sodium [DSS]-induced mice [9, 10]). The most widely accepted animal model of colitis-associated CRC is the AOM/DSS-induced mouse model [11]. In such animal models, growing evidence supports the involvement of the host gut microbiota in initiating and/or perpetuating an immune response, which is a prerequisite for the initiation and development of CRC [12, 13]. For example, it has been reported that bacterial phyla that generate short-chain fatty acids are less frequently observed in patients presenting with IBD; this may be indicative of permanent gut inflammatory responses in IBD; however, whether this is related to cause or effect has yet to be determined [14–16]. Recent findings from a gnotobiotic IL-10^−/−^ mouse model showed that colitis alone is insufficient to facilitate colitis-associated CRC and that an additional, more specific pathobiont is needed, that is, a symbiont capable of promoting CRC pathology only under conditions in which the host's specific genetic or environmental situation is changed [17].

*α*-Ketoglutarate is a crucial component of various cellular metabolic pathways, including regulation of amino acid levels, inhibition of protein catabolism, promotion of protein synthesis, and management of lipid levels [18]. It is a precursor for both glutamine and glutamate and has demonstrated clear clinical benefits, with enhanced immunity resulting in subjects with inflammatory disease or malnutrition [19]. More recently, it was demonstrated that the NF-*κ*B-mediated inflammatory pathway constitutes a homeostatic intestinal control mechanism that circumvents initiation of gut inflammation, which can then lead to eventual tumour formation [19]. However, the actual mechanisms that underlie *α*-ketoglutarate-mediated effects on intestinal inflammation and CRC progression remain poorly understood.

This investigation used the AOM/DSS-induced CRC model to assess whether *α*-ketoglutarate intervention can provide prophylaxis against colitis-associated CRC progression. The antitumour effects of *α*-ketoglutarate were assessed by histopathological analysis and evaluation of the levels of inflammation-associated cytokines. Alterations in intestinal microbiota were determined by 16S rRNA gene sequencing.

## 2. Materials and Methods

### 2.1. Animal and Experimental Treatments

This study was conducted according to the guidelines of the Laboratory Animal Ethical Commission of Hunan Agricultural University. All animal experiments were approval by the Animal Welfare Committee of Hunan Agricultural University. Female C57BL/6 mice (28 d old) were obtained from Hunan SLAC Laboratory Animal Centre, Changsha, China. They were housed in separate sterile animal colonies with controlled temperature and humidity (25°C ± 5°C and 55% ± 5% humidity) and a 12 h dark/light cycle and had free access to standard rodent feed (as in the previous study [20]) and drinking water. The mice were given 3 days to adapt to these conditions before grouping. DSS was obtained from MP Biomedicals (Santa Ana, CA). AOM was obtained from Sigma-Aldrich (St. Louis, MO) and dissolved in normal saline solution to produce a final concentration of 0.5 mg/mL.

After 3 days of adaptation, a total of 24 mice were separated into two experimental groups; the control group received a standard rodent diet, whereas the *α*-ketoglutarate (AKG) group received a standard diet supplemented with 1%  *α*-ketoglutarate. The treatment commenced 1 week before CRC induction and was terminated 1 day before animal sacrifice. Each group initially comprised 12 subjects, which were individually identified and monitored throughout the experiment. Weight measurements were taken on a weekly basis. Intraperitoneal injection of 10 mg/kg of azoxymethane was carried out for CRC induction. One week later, 2.5% DSS was administered via drinking water for 5 days, followed by 14 days of normal drinking water. This regimen was repeated twice, and the subjects were killed 10 days after the final cycle, in line with the procedure described by Greten et al. [21]. Tumour samples were then obtained to evaluate cytokine levels. For serum cytokine analysis, blood samples were also taken. The samples were transferred immediately to liquid nitrogen and stored at −80°C for further analysis.

### 2.2. Histopathological Assessment and Immunohistochemical Analysis

Macroscopic examination of colon biopsies, prepared as per the “Swiss roll” technique [22], was performed to identify tumorigenesis. The tissues were fixed in formalin overnight and transferred to 70% ethanol solution before paraffin-embedding. Paraffin-embedded sections were stained with haematoxylin and eosin (H&E) according to the standard guidelines for histopathological assessment [23].

### 2.3. Cytokines Analysis

Tumour tissue cytokine analysis was conducted using commercial kits (Nanjing Jiancheng Bio-Institute, Nanjing, China) according to the manufacturer's guidelines. Proteins were extracted from tumour tissue using protein extraction buffer and the TissueLyser apparatus at 30 rpm for 3 min. Samples were kept for 20 min and centrifuged at 11,000 rpm for 30 min at 4°C to collect supernatant. The supernatant was then analysed to quantify the protein levels.

### 2.4. High-Throughput Sequencing of 16S rRNA

Extracted mouse colons were dissected longitudinally and washed with precooled phosphate-buffered saline solution (PBS). QIAamp DNA Mini Kit (Qiagen, Hilden, Germany) was used for DNA extraction. Specific PCR (Polymerase chain reaction) primers for the 338-806 (V3-V4) regions (338F, 5′-ACT CCT ACG GGA GGC AGC-3′; 806R, 5′-GGA CTA CHV GGG TWT CTA AT-3′) were used for 16S rRNA PCR amplification. Both preprimers were joined with an Illumina sequencing adapter, while the reverse primer contained a sample barcode. PCR products were purified, and sequencing was performed on an Illumina MiSeq PE300 system (TinyGene Co., Ltd., Shanghai, China). Raw sequence data were demultiplexed using QIIME 1.8.0 [24] and named using sample IDs generated by the QIIME's default quality filtering process [25]. Sequences were apportioned to clusters of 97% similarity by means of QIIME's uclust-based open-reference operational taxonomic unit (OTU) picking protocol against the Greengenes 13_5 reference sequence set [26]. The centroid of each OTU was selected as the representative sequence for the OTU. Statistical significance of alpha diversity variations was determined using nonparametric analysis of variance, and the outcomes were compared among the grouped samples.

### 2.5. Statistical Data Analysis

Numerical data were recorded as mean ± SD. The number of tumours was the primary outcome. The secondary outcomes were gut microbial abundance and diversity, inflammatory index, and cytokine expression. The Mann–Whitney *U* test was used to compare both control and experimental groups for continuous variables, while the chi-square test was used to compare the categorical variables in SPSS 22.0. A *p* value of less than 0.05 was considered to indicate statistical significance.

## 3. Results

**Figure 1:**
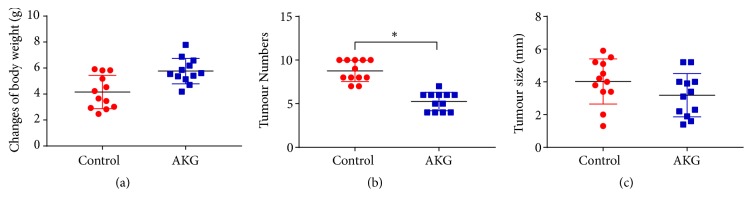
*α*-Ketoglutarate intervention protects the gastrointestinal tracts of the mouse from AOM/DSS-induced CRC: (a) body weight, (b) number and (c) size of colon tumours in the control (*n* = 12) and *α*-ketoglutarate-treated (*n* = 12) groups. *∗ p* < 0.05.

**Figure 2:**
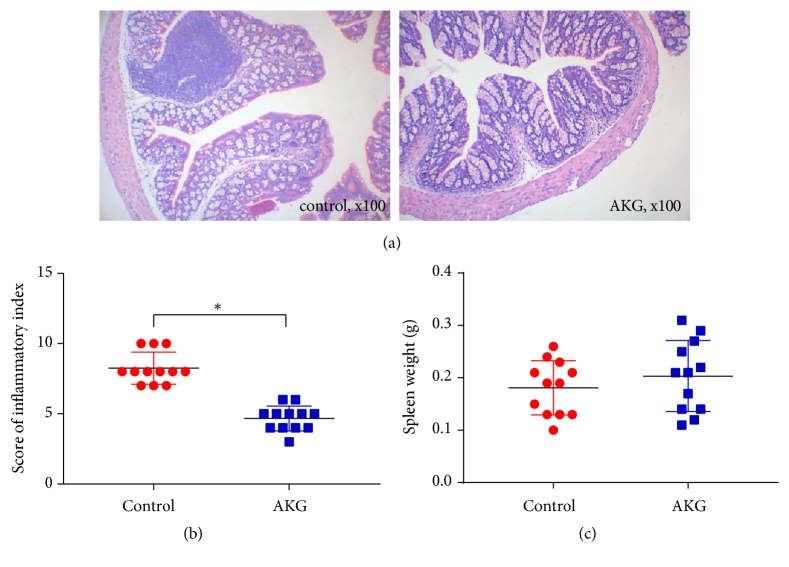
*α*-Ketoglutarate intervention modulates colonic inflammation in CRC: (a) histological imaging, (b) inflammatory index, and (c) spleen weights of the control and AKG-treated mice at 60 days after azoxymethane injection (*n* = 12). *∗ p* < 0.05.

In a colitis-associated CRC model, the development of CRC in mice was studied in relation to *α*-ketoglutarate supplementation. The experimental subjects received intraperitoneal injections of AOM, followed by triplicate treatment cycles with DSS and a 1%  *α*-ketoglutarate dietary supplementation. The body weights of control mice were similar to those of the mice in the AKG-treated group, as depicted in Figure 1(a). Tumour numbers were evaluated at 60 days after AOM injection. The observed tumour numbers were 8.7 ± 1.16 (*n* = 12) for the control group and 5.3 ± 1.01 (*n* = 12) for the AKG-treated group, representing a 39.1% reduction (*p* < 0.05) (Figure 1(b)). However, no significant difference was observed in the mean tumour size between the groups; the AKG-treated group presented moderately smaller tumour sizes compared to the control group (3.2 ± 1.26 vs. 4.0 ± 1.32) (Figure 1(c)). When the inflammatory index of the colonic tissues was assessed, it was found that *α*-ketoglutarate intervention significantly reduced the inflammatory index (*p* < 0.05) but had no effects on spleen weight (Figure 2).

**Figure 3:**
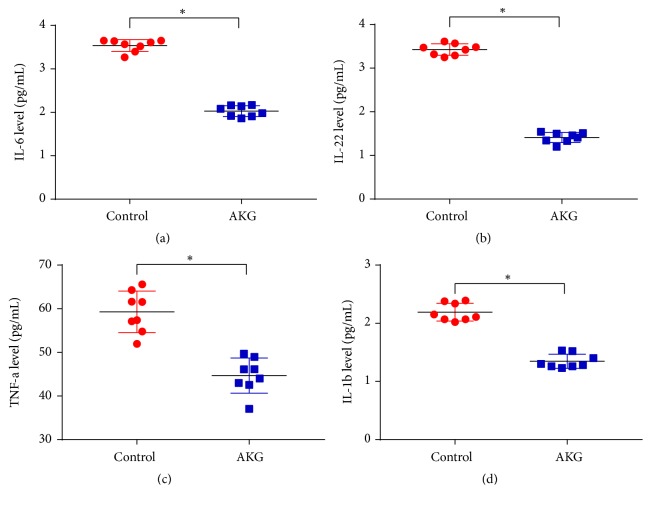
*α*-Ketoglutarate intervention modulates cytokine concentrations: (a) IL-6, (b) IL-22, (c) TNF-*α*, and (d) IL-1*β* protein levels in colon tissues of control and AKG-treated mice at 60 days after azoxymethane injection (*n* = 8). *∗ p *< 0.05.

The cytokine levels in colonic epithelial tissues were evaluated to assess any the influence of *α*-ketoglutarate on anticancer and proinflammatory cytokines in AOM/DSS-treated mice. As shown in Figure 3, *α*-ketoglutarate intervention significantly reduced the concentrations of IL-6, IL-22, TNF-*α*, and IL-1*β* compared to the control group (*p* < 0.05).

**Figure 4:**
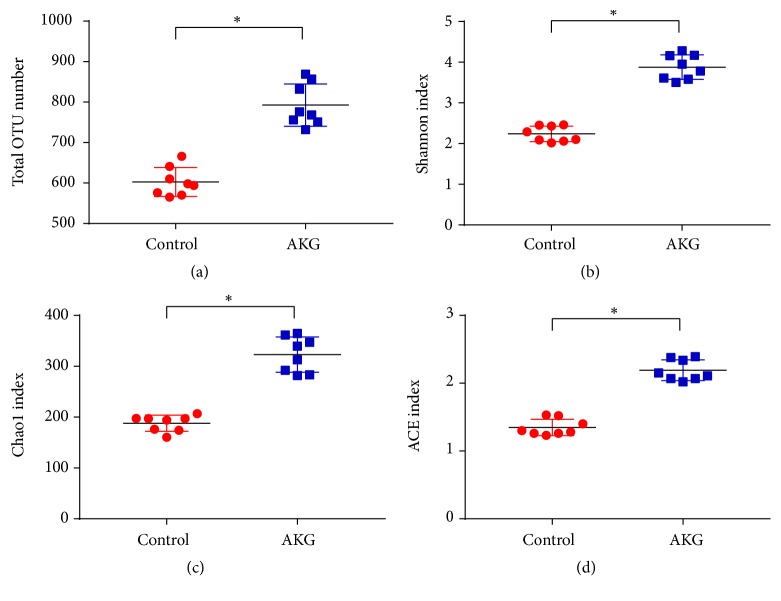
*α*-Ketoglutarate intervention altered the colonic microbial composition: (a) total number of OTUs, (b) Shannon index, (c) Chao1 index, and (d) ACE index of the colonic microbiota at 60 days after azoxymethane injection (*n* = 8). *∗ p* < 0.05.

To investigate the effect of *α*-ketoglutarate intervention on the intestinal microbiome, microbial abundance and diversity were examined. As depicted in Figure 4, the *α*-ketoglutarate intervention increased the total number of microbial OTUs, Shannon index, Chao1 index, and ACE index (*p* < 0.05).

**Figure 5:**
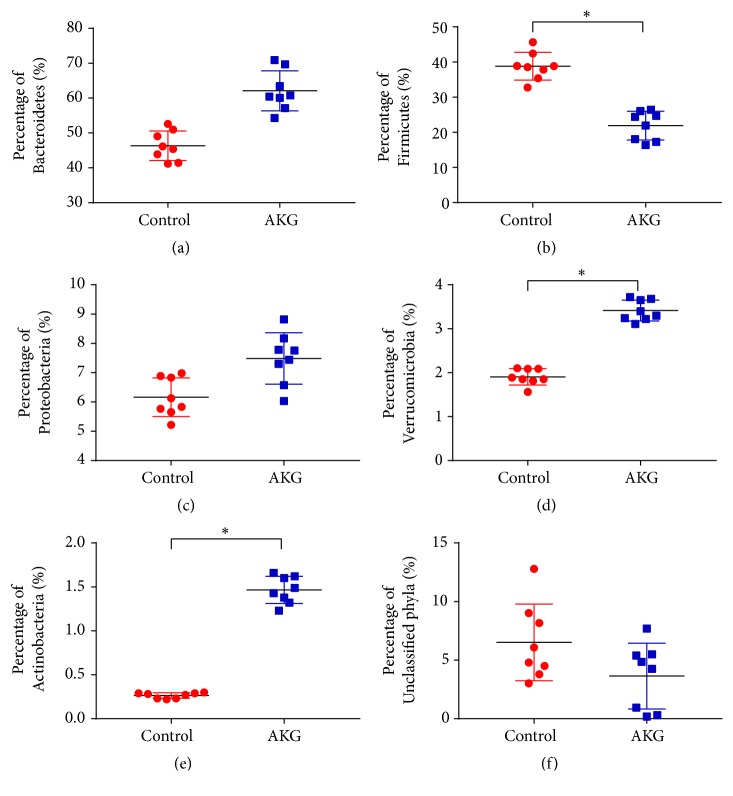
*α*-Ketoglutarate intervention alters the composition of colonic microbiota at the phylum level. Percentage of (a) Bacteroidetes, (b) Firmicutes, (c) Proteobacteria, (d) Verrucomicrobia, (e) Actinobacteria, and (f) unclassified phyla of the colonic microbiota at 60 days after azoxymethane injection (*n* = 8). *∗ p* < 0.05.

**Figure 6:**
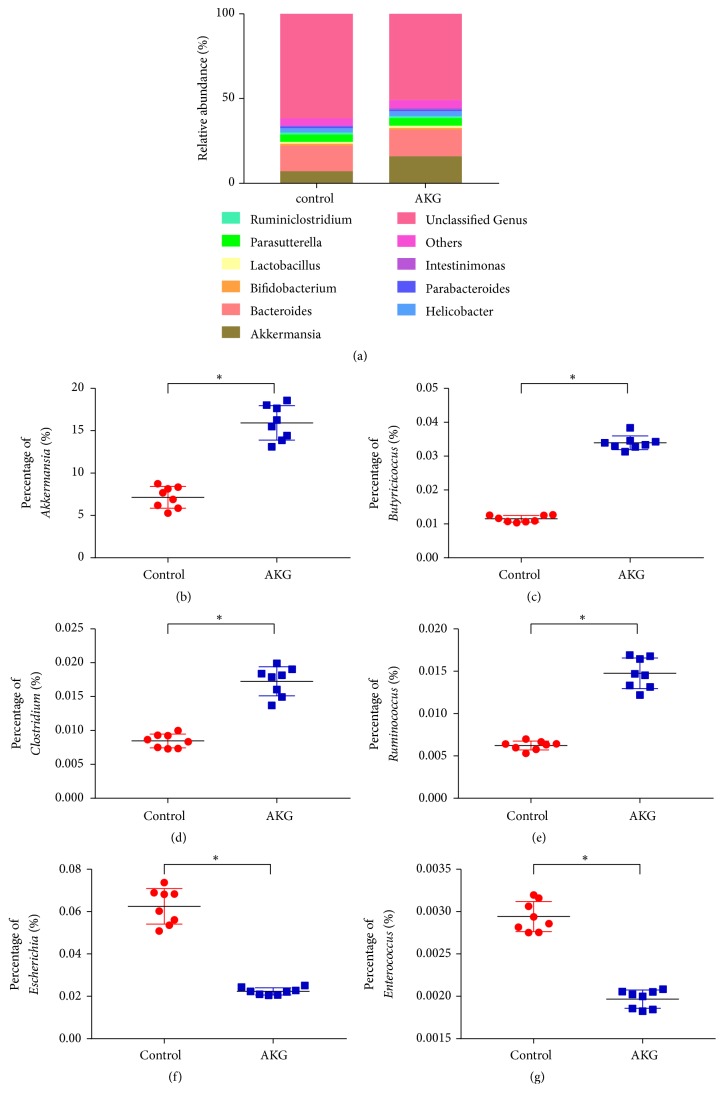
*α*-Ketoglutarate intervention alters the composition of the colonic microbiota at the genus level. (a) Relative percentage of microbiota at genus level and percentage of (b)* Akkermansia*, (c)* Butyricicoccus*, (d)* Clostridium*, (e)* Ruminococcus*, (f)* Escherichia*, and (g)* Enterococcus* of the colonic microbiota at 60 days after azoxymethane injection (*n* = 8). *∗ p* < 0.05.

Bacterial communities were subjected to phylogenetic analysis (Figure 5). Bacteroidetes phylum was the dominant one (46.3 ± 3.96%) in the control group, with Firmicutes ranking second (38.8 ± 3.70%) and Proteobacteria third (6.2 ± 0.62%). Bacteroidetes phylum was also the dominant one in the *α*-ketoglutarate experimental group (62.1 ± 5.37%), with Firmicutes ranking second (21.9 ± 3.84%) and Proteobacteria third (7.5 ± 0.82%). In addition, the *α*-ketoglutarate treatment significantly enhanced the proportion of Verrucomicrobia and Actinobacteria (*p* < 0.05) while reducing the proportion of Firmicutes (*p* < 0.05). At the genus level (Figure 6), the *α*-ketoglutarate intervention significantly increased the proportion of* Akkermansia*,* Bifidobacterium*,* Butyricicoccus*,* Clostridium*, and* Ruminococcus* (*p* < 0.05) while reducing the proportions of* Escherichia* and* Enterococcus* (*p* < 0.05).

## 4. Discussion

A growing body of evidence supports a connection between chronic colonic and rectal damage, as is present in IBD patients, and the initiation of CRC. In fact, in IBD patients, the risk of colitis-associated cancer has been increased [27]. There is now consensus that variation in the composition of the microbiota acts as an initiating step during the progressive development from inflammation to dysplasia to adenocarcinoma. Therefore, prevention of tumour development has been found to be possible via manipulation of the intestinal microbiota [28]. To improve understanding of mechanisms associated with tumour initiation, the AOM-DSS model was used in this study to examine the relationships among imbalances in the microbiota, inflammation, and subsequent CRC development.

Recent studies have reported that AKG intervention has beneficial effects in modulating inflammatory cytokines such as IL-2, IL-8, IL-10, TGF-*β*, TNF-*α*, and IL-17 [29, 30], but excessive production of cytokines has been shown to have a negative effect on intestinal integrity and epithelial restitution [31, 32]. In a lipopolysaccharide (LPS)-induced piglet model, intervention with AKG was shown to inhibit the secretion and mRNA expression of IL-1*β*, IL-6, and IL-12 in the small intestine and prevented tissue damage. Chen et al. reported that AKG intervention in mice reduces body weight and affects the intestinal innate immunity via alterations of gut microbiota [20]. It is interesting that studies have also identified that AKG treatment can inhibit the NF-*κ*B-mediated inflammatory pathway, thereby enhancing gut immunity and promoting self-detoxification systems [29, 33]. It has also been validated that AKG intervention can enhance the expression of inflammatory cytokines [29]. These observations led to the examination of the effects of *α*-ketoglutarate intervention in a murine model of CRC induced by AOM and DSS. The findings demonstrate that intervention with *α*-ketoglutarate reduced the CRC tumour burden in mice and prevented colitis by inducing alterations in gut microbial composition, reducing inflammatory responses within the colon, and modulating cytokine expression. A reduction in IL-22 concentrations was observed in colons from *α*-ketoglutarate-treated mice. It was recently reported that IL-22 is involved in CRC progression in both humans and APC^min/+^ murine model [34]. Therefore, it seems that IL-22 concentrations are increased in tumour cells and that mice exhibiting reduced concentrations of this cytokine are refractory to CRC development.

In this investigation, the alpha diversity of gut microbiota in the colon was evaluated by the Shannon and Simpson indexes, and the richness was detected by the Chao1 and ACE indexes. Significant variation between the control and AKG groups was observed. *α*-Ketoglutarate treatment significantly enhanced the proportion of Verrucomicrobia and Actinobacteria while reducing the proportion of Firmicutes. Of note, Gao et al. reported that the populations of Actinobacteria and Firmicutes were decreased at the phylum level in the intestines of CRC patients [35]. Actinobacteria is one of the key members of genus* Bifidobacterium*, which represents a phylum of gram-positive bacteria [36]. However, treatment with *α*-ketoglutarate enhanced the proportion of this genus in colitis-associated CRC. These findings indicate that *α*-ketoglutarate intervention changes the colitis-associated CRC microbiota to that resembling an anticarcinogenic microbiome.

The increased levels of short-chain fatty acid (especially butyrate)–producing bacteria in the *α*-ketoglutarate treated mice indicate that *α*-ketoglutarate has the propensity to increase the prevalence of some short-chain fatty acid–producing bacteria such as* Butyricicoccus*,* Clostridium*, and* Ruminococcus* in the gut.* Butyricicoccus* has been found to promote intestinal epithelial barrier function and protect gastrointestinal tracts in colitis-associated CRC patients [37].* Clostridium* and* Ruminococcus *were the primary microbes identified in the AKG-treated group, implying that they may be crucial players in the maintenance of normal microbial homeostasis.* Akkermansia muciniphila* is a further example of an intestinal bacterium that may possibly possess anti-inflammatory characteristics in metabolic disorders. The presence of* A. muciniphila* has shown an inverse correlation with cardiometabolic disorders, low-grade inflammatory conditions, obesity, and diabetes [38]. In ulcerative colitis, reduced levels of* A. muciniphila *were observed, but it showed a positive correlation with CRC incidence [39, 40].* A. muciniphila* is a mucin-degrading commensal bacterium that can interrupt the intestinal barrier function, thereby promoting colitis [41]. Conversely, other studies have reported that* A. muciniphila* increases the number of mucogenic goblet cells, thereby promoting repair of the mucus layer [42]. Our study indicates an increased abundance of* A. muciniphila* in the AKG-treated group, which, in combination with other microbes, may have contributed to the prevention of colitis.

To conclude, dietary *α*-ketoglutarate has the potential to offer an effective approach for the prevention of inflammation-associated CRC, irrespective of any anti-inflammatory effects. The incidence of opportunistic pathogens was decreased (*Escherichia* and* Enterococcus*), and the incidence of* Akkermansia, Bifidobacterium, Butyricicoccus*,* Clostridium,* and* Ruminococcus* was increased. Noticeable alterations observed within the microbial community correlated with *α*-ketoglutarate treatment; however, it has not been possible to firmly identify a causal association between these alterations and the reduced carcinogenesis observed. Future in-depth investigations are warranted to examine this hypothesis. Our study demonstrates that *α*-ketoglutarate supplementation may be a viable chemopreventive strategy in IBD patients. Gut microbiota alteration by *α*-ketoglutarate intervention may enhance gut homeostasis and control inflammatory responses, thereby minimising inflammatory cell infiltration via reduced chemokine expression.

## Data Availability

The data used to support the findings of this study are available from the corresponding author upon request.
